# A brief overview of the medicinal and nutraceutical importance of *Inonotus obliquus* (chaga) mushrooms

**DOI:** 10.1016/j.heliyon.2024.e35638

**Published:** 2024-08-06

**Authors:** Emma Camilleri, Renald Blundell, Bikash Baral, Tomasz M. Karpinski, Edlira Aruci, Omar M. Atrooz

**Affiliations:** a Department of Physiology and Biochemistry, Faculty of Medicine and Surgery, University of Malta, Imsida, MSD2080, Malta; b Centre for Molecular Medicine and Biobanking, University of Malta, MSD2080, Imsida, Malta; c University of Helsinki, Helsinki, Finland; h Institute of Biological Resources (IBR), Kathmandu, Nepal; d Department of Medical Microbiology, Poznań University of Medical Sciences, Rokietnicka 10, 60-806, Poznań, Poland; e Western Balkans University, Autostrada Tirane-Durres km 7, Albania; f Department of Medical Laboratory Sciences, Faculty of Applied Medical Sciences, Al-Ahliyya Amman University, Amman, 19328, Jordan; g Department of Biological Sciences, Mutah University, P.O.Box(7), Mutah, Jordan

**Keywords:** Chaga mushrooms, *Inonotus obliquus*, Phytochemicals, Health benefits, Anticancer, Anti-inflammatory, Antimicrobial

## Abstract

This literature review offers an extensive exploration of Chaga mushrooms (*Inonotus obliquus*), focusing on their phytochemical composition, health-promoting attributes, and mechanisms of action. The aim was to provide an up-to-date overview of Chaga's significance in the medicinal sector, emphasizing its potential role in diverse health benefits. The review highlights Chaga's remarkable anticancer, antioxidant, anti-diabetic, anti-inflammatory, antimicrobial, and immunomodulating properties. By synthesizing recent findings, this work underscores Chaga's importance in the medicinal industries and provides valuable insights into its pharmacological potential.

## 1 Introduction

Mushrooms have great potential in the field of modern medicine [1]. Chaga mushroom (*Inonotus obliquus*), a white rot fungus of the Basidiomycetes family Hymenochaetaceae, inhabits birch trees in colder northern regions [2,3], mainly in Siberia, North America, and Scandinavia [[3], [4], [5]]. These mushrooms exhibit a unique appearance characterised by a dark, irregular mass with a charred texture. Chaga mushrooms (CM) have garnered scientific interest for their potential health benefits. With a long history of being used as a folk remedy in Western Siberia and Russia, focus has now shifted to tracing their bioactive constituents, which include polysaccharides [[6], [7], [8]], triterpenoids [9], phenolic compounds, and melanins [[9], [10], [11]]. Traditional medicine has long embraced CM for its presumed immunemodulatory [12,13], antioxidant [14], and anti-inflammatory properties [15,16]. Recent scientific research has started investigating their bioactive compounds and potential therapeutic applications, including anti-cancer, antioxidant, anti-inflammatory, antibacterial, and hepato-protective effects [[17], [18], [19], [20], [21], [22]]as well as support for the immune system [23,24]. Although further investigation is required to fully comprehend their mechanisms of action, CM hold great promise as a subject of scientific exploration for natural health interventions. This article aims to delve into the nutritional composition, bioactive therapeutic components, and potential health benefits of CMs, highlighting their historical use in traditional medicine along with current scientific insights.

## 2 Methodology

### 2.1 Literature search strategy

A comprehensive literature search was conducted to gather relevant studies for this review. Multiple electronic databases, including PubMed, Wanfang, Scopus, Web of Science, and Google Scholar, were used. The search terms used were "Chaga mushrooms," "*Inonotus obliquus*," "phytochemicals of *Inonotus obliquus*," "health benefits of *Inonotus obliquus*," "anticancer effects of *Inonotus obliquus*," "antioxidant action of *Inonotus obliquus*," "anti-diabetic properties of *Inonotus obliquus*," "anti-inflammatory action of *Inonotus obliquus*," "antimicrobial effects of *Inonotus obliquus*," and "immunomodulating effects of *Inonotus obliquus*."

### 2.2 Inclusion and exclusion criteria

Studies included in this review were those that met the following criteria: (a) original research articles, systematic reviews, or clinical trials; (b) focus on the phytochemical composition, health benefits, and mode of action of Chaga mushrooms; (c) provide information on the anticancer, antioxidant, anti-diabetic, anti-inflammatory, antimicrobial, or immunomodulating effects of Chaga; (d) publication in peer-reviewed journals. Studies were excluded if they were (a) non-research articles; (b) not directly related to Chaga mushrooms; (c) lacking relevant information on the health benefits or mechanisms of action; (d) duplicate publications; and (e) not in English.

## 3 Life cycle

CM is a wood-rot fungus colonizing living trees through their wounds, leading to subsequent decay and the formation of a sterile mycelial mass over a period of several years [5]. Following the collapse of the tree, fruiting bodies grow beneath the bark. However, the precise function of the sterile conk [24,25] which isa conk-like growth or structure on the tree's exterior is still unknown. Basidiospores produced by the fruiting bodies mediate the infection process [26], even though the infection may occur through chlamydospores as well [5,27]. CM is mostly found in circumboreal regions in terms of distribution; however, it is still unknown if this refers to a single species with a wide geographic range or several related species. The sterile conks, also known as “chaga” have a long history of traditional use [28]. The hard woody mass is consumed as a tea by boiling and is believed to treat a variety of ailments, including the treatment for cancer, viral [29], bacterial infections, and digestive issues [30,31]. Recent studies on CM's medical uses and health advantages have revealed its powerful anti-cancer [23,32], immune-boosting [33], and antioxidant properties [14,16,34]. CM has thus become one of the most well-investigated medicinal fungi.

## 4 Secondary metabolites of CMs

**Fig. 1:**
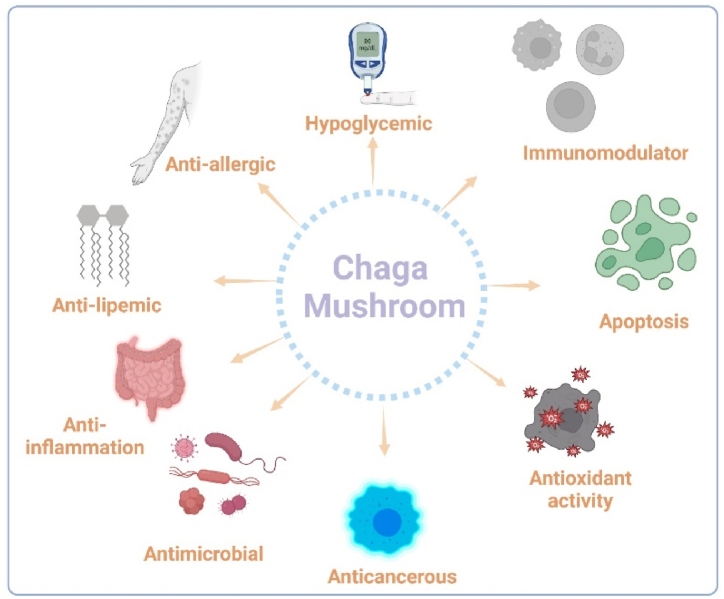
Different activities and roles of secondary compounds present in Chaga mushrooms. As shown CMs have a wide variety of medicinal attributes that is all attributed to their rich and vast phytochemistry.

**Fig. 2:**
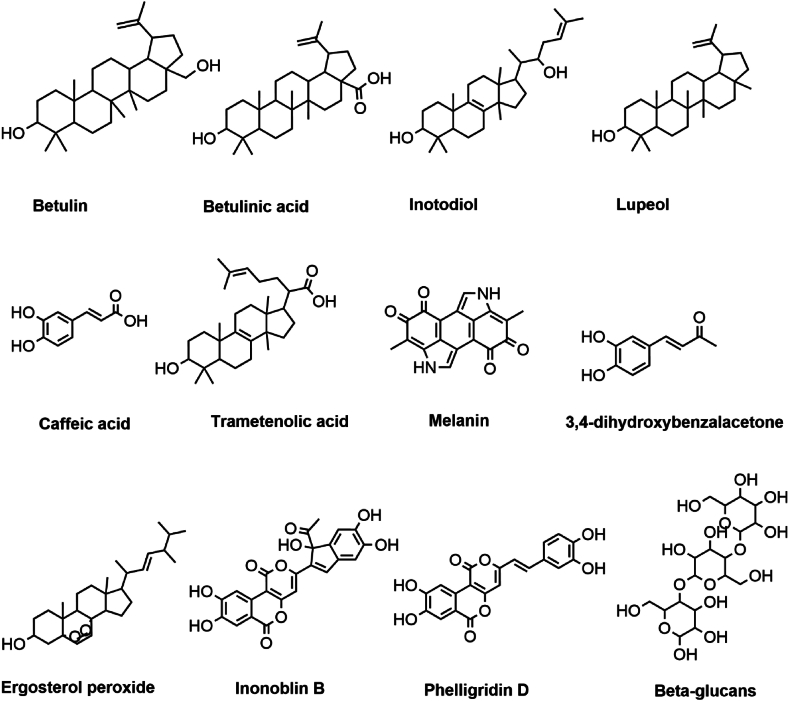
Secondary metabolites present in Chaga mushrooms. It can be appreciated that several phytochemicals within CM contribute to its overall medicinal and industrial potential.

CMs are widely used in conventional medicine and are known to have therapeutic benefits as depicted in Fig. 1 [35]. The mushroom possesses anti-inflammatory, immunomodulatory, antioxidant, antidiabetic and anti-cancer properties [22,[36], [37], [38]]. As seen in Fig. 2 and Table 1, CMs are a viable source of natural products with pharmacological and nutraceutical uses since extracting and isolating these chemicals has demonstrated potential for medication development and therapeutic treatments [6,39]. Due to their varied pharmacological properties, CMs are consumed as a traditional medicine throughout Eastern Europe and Asia, including China, Russia, Korea, and several Western nations [37,40,41]. They include polyphenolic substances such as triterpenoids, steroids, and ergosterol peroxides, which have a variety of biological properties like antimicrobial [42], hepatoprotective [43], and anti-tumour properties [44].

**Table 1:** Biological activities of CMs main components. Several bioactive compounds ranging from polysaccharides to phenols in CM contribute to its pharmacological and therapeutic potential.

Component	Biological activities	References
Polysaccharide and Glucans	Anticancer, anti-inflammatory, antiviral, antioxidant, immunomodulatory, hypoglycemic, hypolipidemic, hepatoprotective, etc.	[8,45,[46], [47], [48]]
Triterpenoid, Lupeol, Lanosterol and Inotodiol	Anticancer, anti-inflammatory, antiviral, and antioxidant	
Melanin	Antioxidant, anti-inflammatory, antiviral, hypolipidemic and immunomodulatory.	
Polyphenol, Phelligridin D, 3,4-dihydroxybenzalacetone, Caffeic acid, Inonoblin B	Anticancer, antioxidant, regulates expression of genes promoting, anti-apoptosis and cell proliferation	
Terpenes, Betulin and Betulinic acid	Antibacterial, protective effects against cadmium induced cytotoxicity, anti-malarial, anti-inflammatory, anti-HIV activities and cyto-toxicity against a variety of tumor cell lines	
Sterol, Ergosterol peroxide, Trametenolic acid	Anticancer, antimicrobial, immunosuppressive	

CMs are rich in triterpenoids, which have been linked to a variety of bioactive qualities. These compounds include betulin, betulinic acid, inonotsuoxides, inotodiol, trametenolic acid, lanosterol [49,50], inonotusols (Kou et al., 2021), spiroinonotsuoxodiol [51], inoterpenes [50], inonotsutriols [52], inotodiol, ergosterol peroxide [24], lanosterol [53,54], and trametenolic acid [4]. Previous studies have reported that triterpenoids in *I. obliquus* have a significant anti-cancer effect on various cancer cells [55,56]. Lupeol is another triterpene found in Chaga with anti-inflammatory and anti-cancer activities. Inotodiol has antiallergic, anti-inflammatory and antiaging acidity, and inhibits cell proliferation and mast cell function [[57], [58], [59], [60], [61]]. Sagayama et al., 2019 suggest that triterpenes found in Chaga mushrooms may promote the proliferation of human follicle dermal papilla cells. Triterpenes have shown protective effects on human keratinocyte cells against oxidative stress and inflammation [62].

Furthermore, *I. obliquus* grows on adult Betula. spp trees and Alnus. *Betula pendula* is a plant with antioxidant and anti-inflammatory properties used in skin diseases [63]. Betula bark oil (*Betulae pix*) has been used for the treatment of skin diseases such as eczema and psoriasis. The birch bark contains about 22 % betulin in its cork tissue. Betulinic acid is another compound found in birch bark and Chaga with antibacterial, anti-malarial, anti-inflammatory, anti-HIV activities and cytotoxicity against a variety of tumour cell lines.

Betulin has anticancer activity [64] and is used for the treatment of wound healing [65,66]. Derivates of betulin stimulate collagen synthesis in normal human fibroblasts [67]. Betulin can be easily converted into betulinic acid, a compound that has anti-malarial, antifungal, anticancer, and anti-inflammatory activity. Betulin and betulinic acid are the most effective compounds used against skin inflammation [68]. Betulin, betunilic acid and their derivates can be used against melanoma skin cancer, epidermoid carcinoma and actinic dermatosis [69]. Yan et al., 2014 studied the effects of betulin, trametenolic, inotodiol and lanosterol on tyrosinase activity and melanin content. In cell testing, betulin and trametenolic acid decreased tyrosinase activity and melanin content, while inotodiol and lanosterol significantly increased tyrosinase activity and melanin content. Ergosterol peroxide has anticancer, antimicrobial and immunosuppressive activity [70].

Polysaccharides such as D-glucans and heteropolysaccharides, among other secondary metabolites, are present in these mushrooms and contribute to their immunomodulatory and anti-inflammatory properties. In addition, polysaccharides can be used in skin-related wound infections [71]. *I. obliquus* polysaccharides have antioxidant activities [72,73]. Du et al., 2014 suggest that β-glucans are promising compounds with wound healing, antioxidant, moisturizing effect and anti-wrinkle activity. Melanin, a pigment with antioxidants [45] and free radical-scavenging properties, is another component of CMs. The chaga mushroom's melanins have been found to reduce nitric oxide production, as well as have antioxidant, genoprotective, and bifidogenic effects according to studies by Wold et al., 2020; Babitskaja et al., 2002 and Burmusova et al., 2019.

Additionally, ergosterol, brassicasterol, and lanosterol are sterols found in CMs that have been associated with anticancer, anti-inflammatory, and immunomodulatory activities [23]. The anti-inflammatory and anticancer activities of Chaga mushrooms are a result of the presence of polyporenic acids [74]. In addition, phenolic substances including protocatechuic acid, caffeic acid, vanillic acid, and syringic acid [75], as well as flavonoids like quercetin, kaempferol [76], and rutin, are present in Chaga mushrooms and support their anti-inflammatory, anti-cancer and antioxidant properties [18,77]. Furthermore, triterpenes of the lanostane class, including inotodiol, are also found and have anti-inflammatory and anticancer properties [78]. Superoxide dismutase (SOD), an enzyme with strong antioxidant activity that guards cells against oxidative stress, is found in CMs [14,79,80].

Some other compounds found in Chaga include isocoumarins, neolignans, cyclic diarylheptanoid (as reported in Chang et al., 2022), alkaloids, amino acids, organic acids and minerals [81]. Additionally, the high oxalate concentration can induce nephropathy. Furthermore, *I. obliquus* can degrade cellulose, hemicellulose, and lignin in the biomass of non-woody plants. Naturally, geographical location, harvesting practices, and processing processes might affect the content and concentration of these compounds. Additional bioactive substances found in CMs are the subject of further study to discover.

## 5 Secondary metabolites having anti-cancer and immunomodulating properties

CMs have gained increased attention for their potential anti-cancer properties [31]. Several compounds found in these mushrooms have been studied for their effects on cancer cells. One of these compounds is betulinic acid, which has shown anti-cancer effects by inducing apoptosis, inhibiting tumour growth, and suppressing metastasis in preclinical models [[82], [83], [84]]. The CM's polysaccharides possess immunomodulatory effects and anti-cancer activities, including enhancing immune responses and inhibiting the proliferation of cancer cells [85]. Chaga polysaccharides have bilateral effects on different cytokines levels such as protein kinase B (Akt) and matrix metalloprotein-9, these cytokines levels were increased in antidiabetic activities but decreased in anticancer effects [86]. Furthermore, Chaga polysaccharides decreased cytokines levels of interleukin (IL)-1B, reactive oxygen species (ROS), and tumour necrosis factor (TNF)-α in antidiabetic activities and increased in anticancer effects [87]. Previous research [37,88] considers the IL-1B and TNF-α are the markers of the activated macrophages (M1) and activated macrophages (M2). These activate M1 and M2 have different transcription profiles and act by eliminating fungi, viruses and bacteria from the host or repairing the damage triggered by inflammation, respectively [89].

Furthermore, triterpenoids, such as inotodiol and ergosterol peroxide, found in CMs have exhibited anti-cancer properties by inducing apoptosis, inhibiting cell proliferation, and suppressing angiogenesis [90,91]. Similarly, the compound called 3,4-dihydroxybenzalacetone has been found to have anticancer properties. It can regulate the expression of genes that promote anti-apoptosis and cell proliferation. Additionally, CMs produce polyporenic acids, including inotolic acid, which have shown promising anti-cancer effects by inhibiting cancer cell growth and inducing cell cycle arrest. The presence of antioxidants, such as melanin, flavonoids, and phenolic compounds, in CMs may also contribute to their potential anti-cancer properties by protecting cells from oxidative stress and DNA damage [9,76]. However, further research, including human clinical trials, is necessary to determine the efficacy and safety of these compounds for cancer treatment. It is important to consult with healthcare professionals before considering CMs as part of a cancer management plan.

## 6 Anticancer activity

Various cancer cell lines, including human colon cancer cells and human hepatoma HepG2 cells, have shown cytotoxic and apoptotic effects in response to CM extract (CME), which have undergone significant research for their anticancer properties. While polysaccharides from Chaga mycelium work by triggering the immune system, those from Chaga sclerotium directly block protein synthesis in malignant cells. These extracts and extracted compounds have shown to be non-toxic and have been used as possible cancer-preventive and chemotherapy substitutes. Endopolysaccharides from the mycelium operate indirectly by stimulating the immune system, whereas heteropolysaccharides and homoglucans from the sterile conk have direct anticancer effects [92]. Chaga contains the triterpenoid inotodiol, which has potent anti-proliferative effects on breast cancer cells. Another substance found in Chaga called ergosterol peroxide works as an antiproliferative agent and prevents colony formation in colon cancer cell lines. It inhibits the -catenin pathway in colorectal cancer, raising the possibility that it may be used to treat the disease [93]. Additionally, the phenolic components in methanolic Chaga extract exhibit specific toxicity against a number of cancer cell lines while sparing healthy cells.

**Fig. 3:**
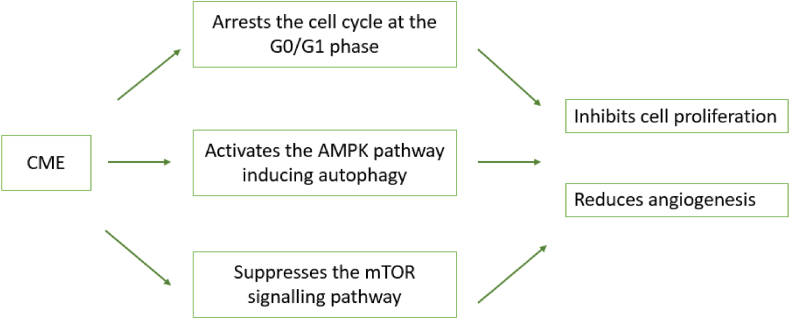
A visual summary of the anticancer activity of CME. As seen in the figure below it can be appreciated that CME prevent cancer cell proliferation by manipulating several pathways.

As shown in Fig. 3, studies revealed the anti-proliferative and apoptotic effects ofCME on a variety of malignancies, including breast, oral, gastrointestinal, lung, and skin cancers [44]. Specific studies with human hepatoma cells (HepG2 and Hep3B) have shown that CME can cause apoptosis and cell cycle arrest in the G0/G1 phase [94]. Additionally, CME has been used as a herbal supplement by cancer patients, including those with triple-negative breast cancer [5,95], and activates autophagy in breast cancer cell lines [96,97]. CME induces autophagy through the activation of AMPK and suppression of the mTOR signalling pathway [4]. As CMs have a wide range of pharmacological effects, including anti-tumour [98] and immunologic capabilities, these are frequently employed in traditional therapies in Eastern Europe and Asia.

Other studies suggest that Chaga extracts can be used in melanoma skin cancer [99], ultraviolet skin protection [63], anti-aging [100] and hyperpigmentary skin disorders. Water extract of Inonotus obliquus mushroom exhibited a potential anticancer activity against B16–F10 melanoma cells in vitro and in vivo through the inhibition of proliferation and induction of differentiation and apoptosis of cancer cells [19]. Harms et al., tested the biological activity of *Inonotus* species on human keratinocytes and showed that these fungi are promising candidates for skin cosmetics too.

## 7 Antioxidant and anti-inflammatory activity

CME have more antioxidant properties than other therapeutic fungi like *Agaricus blazei* mycelia, *Ganoderma lucidum*, and *Phellinus linteus*. Additionally, compared to Chaga decoction, the extract of the fruiting body showed higher antioxidant activity. Thus, CM are renowned for their potent antioxidant properties which are attributed to an array of beneficial compounds [101,102]. These include beta-glucans, phenolics, and melanin, each playing a unique role in cellular protection. Beta-glucans act as cellular shields, scavenging free radicals and preventing damage to healthy tissue [103]. Phenolics neutralize metal ions that may accelerate free radical production, while melanin, the pigment responsible for CM's dark color, also contributes significantly to free radical scavenging. Together, these compounds help protect cells from oxidative stress, which is a major factor in the development of chronic diseases like cancer, diabetes, and heart disease [104,105]. These extracts also have anti-diabetic characteristics because they lower blood glucose levels by inhibiting the enzyme α-glucosidase, an enzyme essential for the breakdown of carbohydrates [106,107]. Additionally, studies on CM polysaccharides reduce blood levels of cholesterol, triglycerides, fatty acids, and glucose [6,79,108]. Furthermore, water-based polysaccharide extract and an ethanol-based extract of Chaga possess notable anti-inflammatory properties, making them interesting candidates for the creation of anti-inflammatory therapeutic drugs.

Generation of pro-inflammatory mediators and cytokines like IL-6, TNF- α, nitric oxide (NO), prostaglandin E2 (PGE2), and IL-1B during inflammation are considered as primary protection of the host [109,110]. It is well-known that inflammation is a physiological immune response of the host body to disease especially injury, chemical toxins, and pathogens and is responsible for the pathogenesis of many diseases [111]. High levels of these mediators lead to oxidative stress and an excessive inflammatory response [112,113]. Macrophages (phagocytic and antigen-presenting cells) play a significant role in the immune system and produce a wide variety of cytokines or markers related to inflammation.

**Fig. 4:**
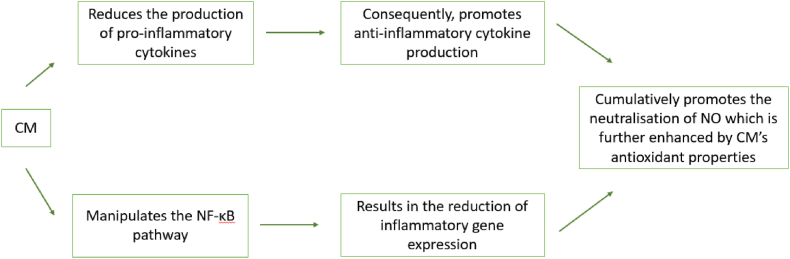
CM's anti-inflammatory activity. It is evident that CM exert its anti-inflammatory properties by influencing several cell pathways.

Research suggests that CM modulate the inflammatory response by regulating cytokine production as seen in Fig. 4. Cytokines are signalling molecules that influence inflammation, with CM reducing the production of pro-inflammatory cytokines such as IL-1β and TNF-α, and potentially enhancing anti-inflammatory cytokines like IL-10 [114]. Moreover, CM appears to influence the NF-κB pathway, a critical regulator of inflammation, thereby potentially decreasing the expression of inflammatory genes [115]. Its antioxidant activity also extends to neutralizing nitric oxide, a molecule involved in the inflammatory process [114]. These multifaceted mechanisms provide a promising basis for CM's potential in managing inflammatory conditions [[115], [116], [117]].

Since the 12th century in Eastern Europe, different traditional preparations of Chaga tea have been used to treat a variety of diseases [118]. In addition, various research has reported that the variety of phenolic compounds in the CME are the main ingredient responsible for its anti-inflammatory and antioxidant activities in vitro and in vivo [119,120]. Other studies demonstrated that polysaccharides, melanin, and polyphenols content in the Chaga extract act as immunomodulating and anti-inflammatory agents. As described, these anti-inflammatory agents produce their effects by regulating pro-inflammatory cytokines and mediators [111,112].

## 8 Antimicrobial activity of Inonotus obliquus

Numerous literature data indicate that extracts from *I. obliquus* show very good antiviral activity. Extracts also have antibacterial and antifungal effects, but this activity is much weaker.

In *in silico* studies, terpenoids found in *Inonotus obliquus* have been found to have the potential to treat SARS-CoV-2. Betulinic acid and inonotusan C can bind to and stably interact with the virus spike protein at the host angiotensin-converting enzyme 2 (ACE2) binding site. Binding affinity values were −7.5 kcal/mol for betulinic acid and −7.4 kcal/mol for inonotusane C [121]. In another *in silico* analysis, betaglycan, betulinic acid and galactomannan, which are components of the Chaga mushroom, were shown to have a strong binding interaction with the SARS-CoV-2 receptor binding domain (RBD). Binding energy values were −8.4 kcal/mol for betaglycan, −8.1 kcal/mol for betulinic acid and −7.4 kcal/mol for galactomannan [122]. The effectiveness of *I. obliquus* water extracts against SARS-CoV-2 is confirmed by the work of Teplyakov et al. Mushrooms collected in Siberia and Altai were used in the research. In cultures on Vero E6 and Vero cells, the activity against the coronavirus strain nCoV/Victoria/1/2020 was very different. IC50 values ranged from 0.75 to 11.6 μg/ml [123].

The water extract of *I. obliquus* shows activity against also the herpes simplex virus (HSV). At a concentration of 3.82 μg/ml, it reduces infection in Vero cells by 50 %. It turns out that the IC50 is >1 mg/ml. The studies also found that the mechanism of anti-HSV action is the prevention of HSV-1 entry and the inhibition of virus-induced membrane fusion [124].

Moreover, water extract of *I. obliquus* inhibits some enzymes, including HIV-1 protease. An extract at a concentration of 2.5 μg/ml has been shown to inhibit HIV-1 protease by 50 %. At a concentration of 70 μg/ml, the extract inhibited the protease by 79 %. In addition, the fraction containing water-soluble lignin derivatives (polyphenols) of high molecular weight leads to the inhibition of HIV-1 protease by 50 % already at a concentration of 1.4 μg/ml. Simultaneously, low-molecular-weight lignin does not inhibit the protease [125]. In the culture of MT-4 lymphoblastoid cells, fractions of water and water-alcoholic extracts of *I. obliquus* have been shown to have antiviral activity against HIV-1 already at a concentration of 5.0 pg/ml [126].

Antiviral activity is also shown by fractions of extracts and melanin found in *I. obliquus.* Fractions of the water extract show virucidal activity against hepatitis C virus (HCV) in porcine embryo kidney cells (SPEV) culture. All extract fractions protected SPEV cells from HCV death at dilutions of 1:4 and above. Some of the fractions show the ability to inactivate the infectivity of the virus, reducing the infectious activity of HCV more than 100 times [127]. Studies indicate that melanin derived from *I. obliquus* has activity against HSV-2, HIV-1, West Nile virus and influenza viruses. Antiviral activity (IC50) against the pandemic H1N1 influenza virus strain is 10–40 μg/ml [128].

It is interesting that ethanol extracts from *I. obliquus* also inhibit animal viruses, including norovirus surrogates murine norovirus (MNV) and feline calicivirus (FCV). It was shown that in cell cultures, the extract at concentrations of 50 and 100 μl/ml reduces the development of FCV at the level of about 40 %. In contrast, the development of MNV is inhibited by approximately 18 % at a concentration of 50 μl/ml of extract and approximately 36 % at 100 μl/ml [129]. Also, polysaccharides from *I. obliquus* exhibit antiviral activity against animal viruses, including feline calicivirus (FCV), feline herpesvirus 1 (FHV-1), feline influenza virus (FIV), feline panleukopenia virus (FPV) and feline infectious peritonitis virus (FIPV). In the case of FCV, polysaccharides at a concentration of 100 μg/ml inhibited the cytopathic effect (CPE) by almost 80 % and at a concentration of 10 μg/ml by 30 %. At the same time, the 50 % inhibitory concentration (IC50) ranged from 25.2 μg/ml to 52.2 μg/ml depending on the virus concentration. The use of polysaccharides significantly reduced viral RNA levels and inhibited viral replication. The IC50 for the other viruses was about 18.2 μg/ml for FHV, 22.9 μg/ml for FIPV, 45.3 μg/ml for FPV and 48.5–68.5 μg/ml for FIV [130].

Water and ethanolic extracts from *I. obliquus* also showed antibacterial activity, however this effect can be described as moderate to weak. The MIC values against Gram-positive bacteria (*Staphylococcus aureus*, *Bacillus cereus*, *Micrococcus flavus* and *Listeria monocytogenes*) ranged between 0.3 and 3.75 mg/ml. A similar MIC range of 0.5–3.75 mg/ml was obtained for Gram-negative bacteria (*Pseudomonas aeruginosa*, *Salmonella typhimurium*, *Escherichia coli* and *Enterobacter cloacae*). At a concentration of 0.5 mg/ml, a reduction of flagella in *P. aeruginosa* was found [131]. Differences in MIC values vary greatly. Taking into account our previous studies, it can be assumed that the activity of extracts against bacteria has medium activity for MICs 0.3–0.5 mg/ml, poor activity for MICs >0.5–1 mg/ml. The values of MIC above 1 mg/ml should be considered as very poor activity or lack of activity (>5 mg/ml) [132,133]. Water and ethanolic extracts of *I. obliquus* also have an antifungal effect, slightly better than against bacteria. MIC levels ranged from 0.2 to 1.5 mg/ml against *Aspergillus fumigatus*, *A. versicolor*, *A. ochraceus*, *A. niger*, *Trichoderma viride*, *Penicillium funiculosum*, *P. ochrochloron* and *P. verrucosum* [131]. The below-presented results are shown in Table 2.

**Table 2:** Antimicrobial activity of Lentinus edodes. Below one can appreciate at which concentrations the bioactive compounds in CM are active in targeting specific pathogens. Thus, it can be appreciated how this remarkable fungus is able to act as an anti-microbial.

Compound/s	Target microorganisms	Active concentration/s	Reference
Terpenoids	SARS-CoV-2 *in silico*	−7.5 kcal/mol for betulinic acid, −7.4 kcal/mol for inonotusane C	[121]
Betaglycan, betulinic acid and galactomannan	SARS-CoV-2 *in silico*	−8.4 kcal/mol for betaglycan, −8.1 kcal/mol for betulinic acid and −7.4 kcal/mol for galactomannan	[122]
Water extracts	SARS-CoV-2	IC50 values ranged from 0.75 to 11.6 μg/ml	[128]
Water extract	Herpes simplex virus (HSV)	IC50 value 3.82 μg/ml	[124]
Water extract	HIV-1	IC50 value 2.5 μg/ml	[125]
Water and water-alcoholic extracts	HIV-1	5.0 pg/ml	[126]
Fractions of the water extract	HCV	dilutions of 1:4 and above	[127]
Melanin	H1N1 influenza virus	IC50 10–40 μg/ml	[128]
Ethanol extracts	Feline calicivirus (FCV)	50–100 μl/ml reduces by 40 % the development of FCV	[129]
Ethanol extracts	Norovirus surrogates murine norovirus (MNV)	50–100 μl/ml reduces by 18–36 % the development of MNV	[129]
Polysaccharides	Feline calicivirus (FCV)	IC50 ranged from 25.2 to 52.2 μg/ml	[130]
Polysaccharides	Feline herpesvirus 1 (FHV-1)	IC50 value 18.2 μg/ml	[130]
Polysaccharides	Feline influenza virus (FIV)	IC50 values 48.5–68.5 μg/ml	[130]
Polysaccharides	Feline panleukopenia virus (FPV)	IC50 value45.3 μg/ml	[130]
Polysaccharides	Feline infectious peritonitis virus (FIPV)	IC50 value22.9 μg/ml	[130]
Water and ethanolic extracts	*Staphylococcus aureus*, *Bacillus cereus*, *Micrococcus flavus* and *Listeria monocytogenes*	0.3 and 3.75 mg/ml	[131]
Water and ethanolic extracts	*Pseudomonas aeruginosa*, *Salmonella typhimurium*, *Escherichia coli* and *Enterobacter cloacae*	0.5–3.75 mg/ml	[131]
Water and ethanolic extracts	*Aspergillus fumigatus*, *A. versicolor*, *A. ochraceus*, *A. niger*, *Trichoderma viride*, *Penicillium funiculosum*, *P. ochrochloron* and *P. verrucosum*	0.2–1.5 mg/ml	[131]

## 9 Conclusion

In conclusion, this review highlights the remarkable potential of CM as a source of diverse health benefits and their significance in the medicinal industry.Through an in-depth exploration of its phytochemical composition and therapeutic benefits, this study underscores Chaga's pivotal role in promoting health and well-being. The examined literature demonstrates the multifaceted properties of Chaga, including its noteworthy anticancer, antioxidant, anti-diabetic, anti-inflammatory, antimicrobial, and immunomodulating effects. These findings suggest that CM possess immense pharmacological potential that could contribute to the development of novel therapeutic interventions.

The amalgamation of research highlights the need for continued investigations into Chaga's bioactive compounds and their intricate interactions with biological systems. As an invaluable natural resource, CM holds promise as a source of bioactive molecules that can contribute to the advancement of medicine and healthcare. Further research is necessary to fully understand Chaga's effects and its integration into mainstream therapeutic approaches.By offering an overview of Chaga's attributes, this review contributes to the understanding of its health benefits and encourages further exploration of its potential applications in both traditional and modern medicine.

## Funding

This research did not receive any specific grant from funding agencies on the public, commercial, or not-for-profit sectors.

## Ethics declaration

Review and/or approval by an ethics committee as well as informed consent was not required for this study because this literature review only used existing data from published studies and did not involve any direct experimentation/studies on living beings.

## Data availability statement

No data was used for the research described in the article. No data associated in this article has been deposited into a publicly available repository.

## CRediT authorship contribution statement

**Emma Camilleri:** Writing – review & editing, Writing – original draft, Visualization, Supervision, Project administration, Methodology. **Renald Blundell:** Conceptualization. **Bikash Baral:** Writing – original draft. **Tomasz M. Karpinski:** Writing – original draft. **Edlira Aruci:** Writing – original draft. **Omar M. Atrooz:** Writing – original draft.

## Declaration of competing interest

The authors declare that they have no known competing financial interests or personal relationships that could have appeared to influence the work reported in this paper.
